# Telehealth: The Way for Efficient, Comprehensive, and Equitable Antimicrobial Stewardship in the US Healthcare System

**DOI:** 10.1093/ofid/ofaf713

**Published:** 2025-12-01

**Authors:** Courtney J Baus, Shivani Patel, Alexander J Lepak, Warren E Rose

**Affiliations:** Department of Pharmacy, University of Wisconsin Hospital and Clinics, Madison, Wisconsin, USA; School of Pharmacy, University of Wisconsin-Madison, Madison, Wisconsin, USA; Department of Pharmacy, Mount Carmel Health System, Columbus, Ohio, USA; Department of Medicine, University of Wisconsin School of Medicine and Public Health, Madison, Wisconsin, USA; School of Pharmacy, University of Wisconsin-Madison, Madison, Wisconsin, USA

**Keywords:** telestewardship, under-resourced communities

## Abstract

Rural and critical access hospitals serve 15% of the United States population and utilize antibiotics at similar rates and spectrum as larger urban hospitals, making them a priority for antimicrobial stewardship. However, barriers such as insufficient personnel, limited electronic health record capabilities, and financial constraints limit stewardship initiatives. Telestewardship partnerships with urban hospitals offer a promising solution; however, a structured process to develop and implement such programs is not established. This perspective focuses on unmet needs in rural hospitals to provide future direction for improved patient care in these settings. In 2024, UW Health engaged leaders of small and rural hospitals to design a telestewardship program that meets regulatory requirements (ie, Joint Commission Standards). Despite these requested services, financial barriers hindered implementation of telestewardship partnerships. This work underscores the opportunities and challenges faced by rural hospitals and the ongoing need for state and national funding to support these communities.

## UNDERSTANDING THE CHALLENGE: ANTIMICROBIAL STEWARDSHIP IN RESOURCE-LIMITED HOSPITALS

Expectations for antimicrobial stewardship in rural and critical access hospitals have increased over the past decade. In 2017, the Joint Commission (TJC) introduced standards for antimicrobial stewardship programs (ASPs) for all hospitals, including critical access hospitals, to maintain accreditation [1]. At this time, the Infectious Diseases Society of America released a position statement supporting telemedicine to provide optimized care to resource-limited populations [2]. In 2019, the Centers for Medicare and Medicaid Services (CMS) enacted the Omnibus Burden Reduction Final Rule, mandating ASPs for reimbursement [3]. Most recently, effective 1 January 2023, TJC expanded stewardship standards (12 elements of performance in the “Medication Management” chapter Standard MM.09.01.01) for all accredited hospitals [4].

These updated standards require hospitals to establish antibiotic stewardship as an organizational priority by allocating financial resources and appointing infectious diseases (ID) or stewardship-trained physician or pharmacist leaders. They require evidence-based guideline implementation, tracking and reporting of antimicrobial use, implementation of preauthorization, prospective review and feedback, and more [4]. Compliance and effectiveness remain difficult for lower-resourced institutions due to inadequate staffing, limited electronic health record (EHR) capabilities, and associated cost of implementation. First, there is a shortage of ID-trained physicians, pharmacists, and infection control personnel nationwide [5]. Ongoing personnel shortages, combined with known recruitment and retention challenges at rural institutions, result in nearly half of smaller hospitals functioning without an ID specialist [5, 6]. Multiple studies demonstrate the benefit of ID consultation on patient outcomes, and its absence risks unnecessary patient complications [7, 8]. Second, successful ASPs rely on data tracking and reporting to identify inappropriate prescribing and programmatic impact. Although EHRs are now standard among hospitals in the United States (US), the capabilities of these systems at rural and critical access hospitals are limited. A study evaluating Centers for Disease Control and Prevention core elements noncompliance in critical access hospitals found EHR system limitations to be among the 3 greatest reported barriers to compliance [9]. Finally, it is costly to hire specialists and expand the technological support necessary for effective ASPs.

Despite these challenges, the need for effective ASPs is evident, given that small and critical access hospitals utilize antibiotics at a rate and spectrum similar to that of large urban hospitals [10]. Significant healthcare disparities exist for patients in these settings compared to their urban counterparts with access to established ASPs [11]. An innovative approach is leveraging telehealth technology, which has demonstrated value within ID since the coronavirus disease 2019 pandemic [12]. Partnerships between academic medical centers and small community hospitals demonstrate improved clinical outcomes and lower utilization of broad-spectrum antibiotics [10, 13–15]. The Duke Antimicrobial Stewardship Outreach Network (DASON) demonstrated successful partnership with community hospitals across the Southeast. However, despite its longstanding history and grant funding, the costs of sustained partnership remain a challenge [16, 17] Effective ASPs are essential for reducing antimicrobial resistance and improving patient safety [7, 8]. Telestewardship can ensure that all hospitals, and their patients, have effective antimicrobial stewardship strategies in place for equitable care.

## DEVELOPMENT OF A TELESTEWARDSHIP MODEL TO SUPPORT REGIONAL RURAL HOSPITALS

Rural and critical access hospitals serve roughly 15% of the US population; however, this varies regionally. In Wisconsin, nearly 30% of hospitals are designated as critical access [18]. In 2015, UW Health piloted a telestewardship partnership with a 95-bed community hospital in Wisconsin. The service included prospective audit and feedback, guideline and order set management, and staff education. The 3-stage pilot demonstrated significant reduction in broad-spectrum antibiotic use and decreased length of stay during the intervention phase compared to both pre- and postintervention phases [13]. For example, imipenem use decreased by 62.7% (*P* < .0001), levofloxacin use decreased by 19.7% (*P* < .0001), piperacillin-tazobactam use decreased by 7.1% (*P* = .12), and vancomycin use decreased by 18.0% (*P* < .0001). A total of 244 audit and feedback interventions were made, with high rates of acceptance by the primary clinicians (eg, 74% of de-escalation and 88% of modification of duration suggestions were accepted). For several infection types, length of stay and mortality trends were lower in the intervention group. The partnership, while highly successful and very well received based on clinician surveys, was not continued due to limited fiscal resources to support the full-time equivalent required.

In 2024, UW Health's inpatient antimicrobial stewardship leaders sought to revitalize our telestewardship program to support critical access hospitals in rural Wisconsin and northern Illinois, aiming to provide specialized stewardship while maintaining localized care. We developed a tier-based customizable telestewardship program based on 2023 TJC requirements and previous experience from our cost-effective pilot program. The base consultative tier encompasses incorporating guideline and protocol adaptations, formulary review, and quarterly assessment and impact reports, while the most comprehensive tier expands to include prospective audit and review, continuing education, direct ID physician consultation, and antimicrobial use tracking and reporting (Table 1). Tier selection is made by the partner institution prior to program implementation following institutional needs assessment and discussion on stewardship goals. This remote telestewardship program is designed to primarily utilize secured messaging platforms within an EHR and as-needed phone-based consultations, with the ability for onsite visits periodically as needed. Licensure and credentialing requirements for healthcare personnel providing these services regionally outside of their states were not addressed in these discussions.

**Table 1.** Tier and Customization Options for Telestewardship Services in Rural Hospitals

	Comprehensive	Partnership	Consultative
Summary	Full-service stewardship, leadership^a^, systematic assessments, implementation support, daily patient review, formulary restriction	Access to experts, systematic program assessment, implementation support, guidance, education	Expert consultation, systematic program assessment, access to clinical practice tools
Program impact	Most impact	High impact	Meets accreditation standards
Quarterly program assessment and action report	Yes	Yes	Yes
Formulary review and recommendations	Yes	Yes	Yes
Access to current UW Health guidelines/protocols	Yes	Yes	Yes
Support with adapting and implementing current UW Health guidelines and protocols	Yes	Yes	No
Continuing education programming to support AMS	Yes	Yes	No
Analyze and report data on AMS initiatives	Yes	Yes	No
Pharmacist-led prospective auditing and data review^b^	Yes	No	No
Direct access to ID-trained physicians	Yes	No	No
Patient- and family-centered education resources	Yes	No	No
National Healthcare Safety Network reporting	Yes	No	No
Implement AMS improvement initiatives^c^	Led by UW Health	Led by institution	Suggestions provided
Microbiology lab optimization^d^	Led by UW Health	Led by institution	Suggestions provided
Prior authorization/restricted antimicrobial program	Available at an additional fee	Available at an additional fee	Available at an additional fee

Abbreviations: AMS, antimicrobial stewardship; ID, infectious diseases.

^a^If necessary. If program is already in place with physician and/or pharmacist leaders assigned, UW Health role will be supportive.

^b^Requires EPIC software system 2015 and commitment to build and support minimally invasive stewardship interventions and tracking.

^c^Examples include antibiotic order form, indications, pharmacokinetic/pharmacodynamic and renal dose optimization strategies, route interchange, antibiotic allergy.

^d^Rapid diagnostics, cascade reporting.

We met with directors of pharmacy and clinical pharmacy coordinators involved in stewardship efforts from 4 potential partner hospitals to understand their current practice and discuss partnership strategies. Two of these hospitals were critical access with ≤25 beds, and the other two were both rural acute care hospitals with bed sizes ranging from 95 to 200. Generally, the extent of stewardship efforts was limited to implementation of a few clinical guidelines, while one institution was just starting the process of developing a stewardship role.

These discussions provided significant insight into the needs and expectations for a telestewardship program. The most requested service was access to an ID-trained physician for on-call consultations. Secondary priorities included guideline and protocol access and expert assistance identifying stewardship targets. Unsurprisingly, the primary challenge identified was cost. While telestewardship studies demonstrate long-term cost savings from reduced antimicrobial utilization and decreased hospital patient-days, these cost benefits typically take 1–2 years to materialize [19]. Unfortunately, stewardship efforts are not currently revenue generating, which is financially preferred by hospitals. Another barrier identified was the lack of informatic capabilities to assess antimicrobial utilization, limiting the ability to identify high-priority stewardship targets and gauge program success. The most eye-opening takeaway from these discussions was the variability in interpretation of the CMS requirement “An individual (or individuals), who is qualified through education, training, or experience in infectious diseases and/or antibiotic stewardship, is appointed by the governing body as the leader(s) of the antibiotic stewardship program.” Many rural centers feel they meet CMS requirements; however, through these conversations we identified that often the individual carrying out delegated stewardship work had limited, if any, stewardship training and background. This in turn, explains that the primary service of interest is partnership with our center for ID physician and pharmacist expertise. We surmise there is a disconnect between the scope of stewardship regulatory requirements and what these smaller health centers currently provide. In our opinion, stronger guidance or policies requiring ID-trained physicians and pharmacists to lead, or direct in advisory roles, is warranted. This would significantly strengthen ASPs at smaller health centers and provide a supportive mandate for resource allocation, while also meeting their most pressing self-identified needs.

## A WAY FORWARD: USING STRATEGIC STEWARDSHIP SUPPORT TO OPTIMIZE RURAL HEALTH RESOURCES

**Figure 1.**
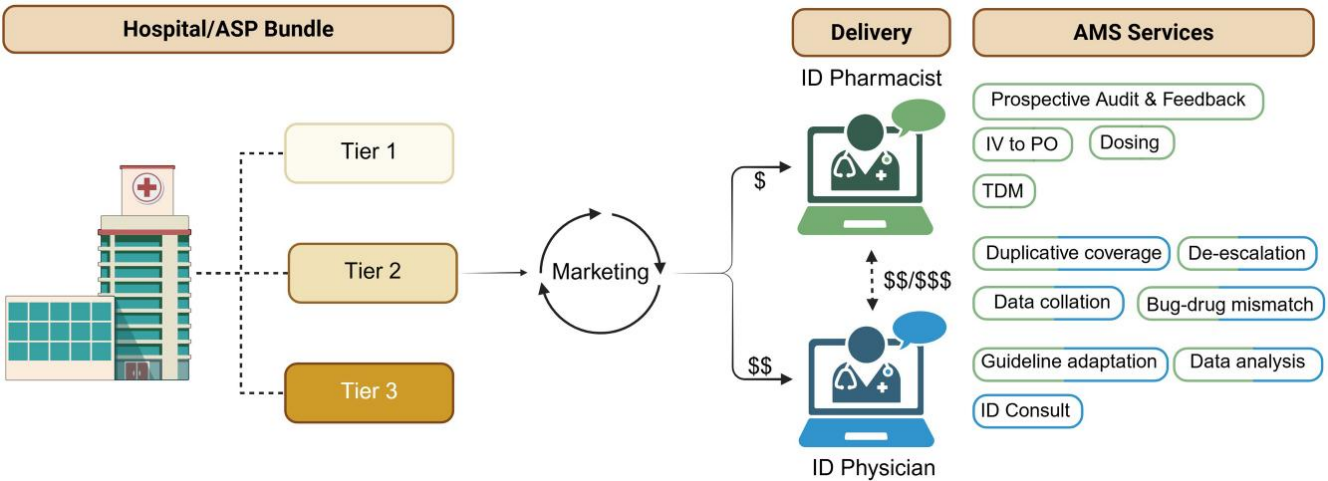
Model for telestewardship implementation for rural, critical access hospitals. Graphic created in BioRender. For antimicrobial stewardship services, green ovals indicate delivery of the service by a pharmacist, blue indicates physician delivery, and half green/half blue ovals indicate shared services. Abbreviations: AMS, antimicrobial stewardship; ASP, antimicrobial stewardship program; ID, infectious diseases; IV, intravenous; PO, oral; TDM, therapeutic drug monitoring.

In lieu of changing stewardship regulatory directives and policies, we continue to strategize how to provide stewardship support while minimizing cost. Physician hours, while the most desired by our cohort, are the highest hourly cost service. To offset physician workload, our program utilizes ID pharmacists for prospective audit and feedback, allowing early identification of complex patients who would benefit from direct ID physician involvement, while reducing total physician hours (Figure 1). ID-trained pharmacists can also evaluate intravenous-to-oral switches, duplicative coverage, de-escalation, dosing optimization, therapeutic drug monitoring, and basic drug–bug mismatches. ID pharmacists can lead guideline adaptation, data gathering, and initial antimicrobial utilization analysis. We note that full-time equivalent allocation for pharmacists/physicians is an important aspect, and that the financial compensation (fee) for the program must align with the effort expected to be needed to provide the services. The latter highly depends on capabilities of the pharmacist and physician, the bed size, complexity of patients, and EHR systems, among other variables. Another approach to mitigate expense to individual hospitals is offering services to multiple hospitals simultaneously via sharable cost, decreasing individual expense while maintaining access to expert stewardship support. While cost-sharing may come with its own challenges, it is prudent to consider. The Rural Wisconsin Health Cooperative is an example of a collaborative network designed to support rural organizations and provide affordable strategies to improve care. Partnership with organizations such as this may be a way to bridge the gap. We acknowledge, despite these strategies, that cost may remain a barrier that limits telestewardship. If so, more efforts toward financial impact and resources are needed. For example, strengthening and delineating TJC/CMS mandates to specify how the resources should be allocated for stewardship could optimize this resource. Another approach is to enhance funding opportunities at federal and state levels to allow programs an opportunity to demonstrate benefit and cost savings prior to transitioning into a mutually beneficial sustainable partnership.

Despite barriers, our programmatic goal remains to expand the reach of antimicrobial stewardship to rural hospitals across Wisconsin and northern Illinois with a focus on antimicrobial optimization, reduced antimicrobial resistance and expenditures, and improved patient outcomes.

## CONCLUSIONS

Stewardship has advanced across the healthcare enterprise over the last decade, but disparities still exist in rural and critical access hospitals. Resource constraints in these settings present significant barriers for optimal ASP implementation. Mitigating urban-rural disparities, optimizing resource utilization, and maximizing effectiveness are paramount principles in healthcare. We outline and propose pharmacist-driven telestewardship with ID physician support to target high-value stewardship goals and reduce the initial hurdles for effective ASP implementation in rural hospital settings.
